# Supplementary material for: Validity of acute cardiovascular outcome diagnoses in European electronic health records: a systematic review protocol
Source: BMJ Open. 2019 Oct 18;9(10):e031373. doi: 10.1136/bmjopen-2019-031373 (PMC6803089; doi:10.1136/bmjopen-2019-031373)
Supplement: Supplementary data [file bmjopen-2019-031373supp001.pdf]

Appendix 1. Provisional MEDLINE search terms

1 (Europe or Albania or Andorra or Armenia or Austria or Azerbaijan or Balkan* or Belgium or Belarus or Byelarus or Belorussia or Bosnia or Herzegovia or Bulgaria or Croatia or Cyprus or Czechoslovakia or Czech Republic or Denmark or Faeroe Islands or Estonia or Finland or France or Germany or Great Britain or GBR or United Kingdom or UK or (Wales not New South Wales) or (England not New England) or Northern Ireland or Scotland or Channel Islands or Isle of Man or Greece or Gibraltar or Hungary or Iceland or Ireland or Eire or Italy or Latvia or Liechtenstein or Lithuania or Luxembourg or Kosovo or Macedonia or Malta or Mediterranean or Moldova or Monaco or Montenegro or Netherlands or Holland or Norway or Poland or Portugal or Romania or Russia or Russian Federation or USSR or "Union of Soviet Socialist Republics" or Soviet Union or San Marino or Scandinavia or Serbia or Slovakia or Slovak Republic or Slovenia or Spain or Balearic Islands or Canary Islands or Sweden or Switzerland or Ukraine).ti,ab.
2 turkey.ti,ab. not animal/
3 exp Europe/
4 or/1-3
5 exp Stroke/ or exp Brain Infarction/ or exp Cerebral Hemorrhage/ or exp Subarachnoid Hemorrhage/ 6 stroke.ti,ab.
7 cerebrovascular accident.ti,ab.
8 ((brain* or cerebr* or intracerebral or intracran* or subarachnoid) adj (infarct* or thrombo* or emboli* or h?emorrhage or h?ematoma or bleed*)).ti,ab.
9 or/5-8
10 exp Myocardial Infarction/ or Acute Coronary Syndrome/
11 (myocardial infarct* or MI or AMI or acute coronary syndrome or ACS).ti,ab.
12 ((cardiac or heart) adj (infarct* or attack* or arrest* or event*)).ti,ab.
13 (stemi or st-segment or st segment or st-elevat* or st elevat*).ti,ab.
14 (nstemi or non-st-segment or non-st segment or non st segment or non-st-elevat* or non-st elevat* or non st elevat*).ti,ab.
15 or/10-14
16 exp Heart Failure/
17 (heart failure or cardiac failure or CCF or left ventricular failure).ti,ab.
18 (left ventricular adj (systolic or diastolic) adj (dysfunction or impairment)).ti,ab.
19 or/16-18
20 9 or 15 or 19
21 Hospital Records/ or exp Medical Records/
22 ((electronic* or digital* or computer* or longitudinal) adj (health* or medical or clinical or patient) adj (record* or data* or regist*)).ti,ab.
23 (EHR or EPR or EMR or EPD).ti,ab.
24 (routine* collected adj2 data).ti,ab.
25 ((primary care or general practice or secondary care or hospital* or health* or administrative or automated) adj2 (record* or data* or regist*)).ti,ab.
26 exp Clinical Coding/ or exp International Classification of Diseases/
27 ((clinical or medical or read or OXMIS) adj cod*).ti,ab.
28 SNOMED.ti,ab.
29 (International Classification of Diseases or ICD10* or ICD-10* or ICD9* or ICD-9* or International Classification of Primary Care or ICPC).ti,ab.
30 (Clinical Practice Research Datalink or CPRD or General Practice Research Database or GPRD or Value Added Medical Products or VAMP or Hospital Episode Statistics or HES or The Health Improvement Network or QResearch or ResearchOne or Danish National Patient Registry or DNPR or Intego or National Patient Register or NPR or patientregistret or National Inpatient Register or IPR or slutenvardsregistret or Finnish Hospital Discharge Register or FHDR or BIFAP or DIRAYA or

Dutch National Basic Registration of Hospital Care or Landelijke Basisregistratie Ziekenhuiszorg or LBZ or Dutch Hospital Discharge Register or Landelijke Medische registratie or LMR or Mondriaan or Netherlands Primary Care Research Database or NPCRD or Integrated Primary Care Information or IPCI or Information Network of General Practice or LINH or French Hospital Discharge Database or FHDDB or Health Longitudinal Patient Database or HSD).ti,ab.
31 or/21-30
32 Validation Studies/ or exp Reproducibility of Results/ or exp "sensitivity and specificity"/
33 (sensitivity or specificity or positive predictive value or PPV or negative predictive value or NPV).ti,ab.
34 (accura* or consisten* or reliab* or reproduc* or complet* or precis* or concordance or variation or variab* or replicat* or verif* or valid* or predict*).ti,ab.
35 (ROC or receiver operating characteristic or kappa).ti,ab.
36 or/32-35
37 4 and 20 and 31 and 36
