# Supplementary material for: Validity of acute cardiovascular outcome diagnoses in European electronic health records: a systematic review protocol
Source: BMJ Open. 2019 Oct 18;9(10):e031373. doi: 10.1136/bmjopen-2019-031373 (PMC6803089; doi:10.1136/bmjopen-2019-031373)
Supplement: Author's manuscript [file bmjopen-2019-031373.draft_revisions.pdf]

BMJ Open is committed to open peer review. As part of this commitment we make the peer review history of every article we publish publicly available.

When an article is published we post the peer reviewers' comments and the authors' responses online. We also post the versions of the paper that were used during peer review. These are the versions that the peer review comments apply to.

The versions of the paper that follow are the versions that were submitted during the peer review process. They are not the versions of record or the final published versions. They should not be cited or distributed as the published version of this manuscript.

BMJ Open is an open access journal and the full, final, typeset and author-corrected version of record of the manuscript is available on our site with no access controls, subscription charges or pay-per-view fees (http://bmjopen.bmj.com).

If you have any questions on BMJ Open's open peer review process please email info.bmjopen@bmj.com

BMJ Open

The validity of acute cardiovascular outcome diagnoses in European electronic health records: a systematic review protocol

Journal:	*BMJ Open*
Manuscript ID	bmjopen-2019-031373
Article Type:	Protocol
Date Submitted by the Author:	30-Apr-2019

Complete List of Authors:	Davidson, Jennifer; London School of Hygiene and Tropical Medicine, Faculty of Epidemiology & Population Health<br>Banerjee, Amitava; University College London, Farr Institute of Health Informatics Research<br>Muzambi, Rutendo; London School of Hygiene and Tropical Medicine, Faculty of Epidemiology & Population Health, London<br>Smeeth, Liam; London School of Hygiene and Tropical Medicine, Faculty of Epidemiology & Population Health<br>Warren-Gash, Charlotte; London School of Hygiene and Tropical Medicine, Faculty of Epidemiology & Population Health

Keywords:	electronic health records, validation, systematic review, cardiovascular diseases

SCHOLARONE™
Manuscripts

Title: The validity of acute cardiovascular outcome diagnoses in European electronic health records: a systematic review protocol

Authors: Jennifer A. Davidson[1], Amitava Banerjee[2], Rutendo Muzambi[1], Liam Smeeth[1], Charlotte Warren-Gash[1]

Author affiliations:
1 London School of Hygiene and Tropical Medicine, Faculty of Epidemiology & Population Health, London, UK
2 University College London, Farr Institute of Health Informatics Research, London, UK

Corresponding author details

Name: Jennifer Davidson
Postal address: London School of Hygiene and Tropical Medicine, Room LG21, Keppel Street, London, UK, WC1E 7HT
Email address: Jennifer.Davidson@lshtm.ac.uk
Phone number: +44 (0)20 7636 8636

Key words: electronic health records; cardiovascular diseases; validation; systematic review

Word count: 1,979

For peer review only

For peer review only - http://bmjopen.bmj.com/site/about/guidelines.xhtml

Abstract

Introduction Cardiovascular diseases (CVDs) are among the leading causes of death globally. Electronic health records (EHRs) provide a rich data source for research on CVD risk factors, treatments and outcomes. Researchers must be confident in the validity of diagnoses in EHRs, particularly when diagnosis definitions and EHRs use change over time. Our systematic review provides an up-to-date appraisal of the validity of stroke, acute coronary syndrome (ACS) and heart failure (HF) diagnoses in European primary and secondary care EHRs.

Methods and analysis We will systematically review published and grey literature to identify studies validating diagnoses of stroke, ACS and HF in European EHRs. MEDLINE, EMBASE, SCOPUS, Web of Science, Cochrane library, OpenGrey and EThOS will be searched from dates of inception to April 2019. A prespecified search strategy of subject headings and free text terms in the title and abstract will be used. Two reviewers will independently screen titles and abstracts to identify eligible studies, followed by full-text review. We require studies to compare clinical codes with a suitable reference standard. Additionally, at least one validation measure (sensitivity, specificity, positive predictive value or negative predictive value) or raw data, for the calculation of a validation measure, is necessary. We will then extract data from the eligible studies using standardized tables and assess risk of bias in individual studies using the Quality Assessment of Diagnostic Accuracy Studies 2 (QUADAS-2) tool. Data will be synthesised into a narrative format and heterogeneity assessed. Meta-analysis will be considered when a sufficient number of homogenous studies are available. The overall quality of evidence will be assessed using the Grading of Recommendations, Assessment, Development and Evaluation (GRADE) tool.

Ethics and dissemination This is a systematic review so does not require ethical approval. Our results will be submitted for peer-review publication.

PROSPERO registration number CRD42019123898

For peer review only

For peer review only - http://bmjopen.bmj.com/site/about/guidelines.xhtml

ARTICLE SUMMARY

Strengths and limitations of this study

- This systematic review will comprehensively evaluate the validity of selected major cardiovascular (CV) diagnoses (stroke, acute coronary syndrome and heart failure) in primary and secondary care electronic health records (EHRs).
- High quality future research on CV conditions using EHRs depends on accurate case classification; any gaps in this which we identify will inform the scope and interpretation of EHR research studies.
- This review focuses on validation methodology and results; it is likely that the included studies will differ in the reference standard used for validation and some that do not find positive results are less likely to have been published.

INTRODUCTION

Rationale

Stroke, acute coronary syndrome (ACS) and heart failure (HF) are three cardiovascular (CV) conditions which substantially contribute to morbidity and mortality. Ischaemic heart disease followed by stroke have been the global leading causes of death for 15 years, and in 2016 accounted for 15.2 million deaths (1). Also in 2016, worldwide more than 13 million people are estimated to have suffered a stroke (2) with healthcare expenditure on stroke estimated to be 3-5% (3–5). An estimated 26 million people are living with HF (6), a chronic condition with acute episodes. HF is estimated to account for 1-2% of healthcare expenditure in Europe and the USA (7). Added to the complication of estimating the burden of CV conditions is changes to definitions; the fourth universal definition of myocardial infarction (MI) was issued in 2018 (8).

Increases in the incidence and prevalence of CV conditions is in part due to an ageing population (9), but also due to modifiable risk factors such as smoking, unhealthful diet and lack of physical exercise, and other non-modifiable risk factors such as sex and ethnicity (10). A range of factors such as pollution, infections, emotional stress and physical exertion can also trigger acute CV events particularly in those with pre-existing cardiovascular disease (CVD) (11–13).

Electronic health record (EHR) databases with longitudinal patient data provide rich data sources for a wide scope of research on CV risk factors as well as treatment and outcomes. The availability of EHRs has increased over time (14) and can be drawn from various sources including primary and secondary care clinical systems, disease registers, and claims and billing databases. Each of which have a different primary non-research purpose. The quality of EHRs based on clinical care systems is directly related to recording practices over time or between staff members which can be influenced by financial incentive schemes. The data within EHRs provide a method of reviewing if healthcare providers meet targets for financial incentives. For example, in the UK, the Quality and Outcomes Framework scheme introduced in 2004 offers general practitioners rewards for managing patients with a range of clinical conditions and has improved the recording of related data in primary care EHRs (15).

Therefore, the use of EHRs as a reliable data source for CV research depends upon the ability of accurately identifying and classifying cases and non-cases. Several older systematic reviews have previously appraised the validation of specific EHRs (16,17) as well as specific conditions recorded within EHRs, including CV conditions (18–22). The previous systematic reviews on the validity of CVD

For peer review only - http://bmjopen.bmj.com/site/about/guidelines.xhtml

diagnoses in EHRs included all data sources (i.e. claims and clinical care data) and produced mixed results. For example, McCormick *et al* reported that the positive predictive value (PPV) of stroke diagnosis ranged from 32-98%, with the majority of included studies using administrative health data from North America (21), while Woodfield *et al* identified PPVs of >70% for stroke based on results which included a greater proportion of studies from Europe (19). McCormick *et al*'s review of HF diagnosis validity obtained PPVs ranging from 17-100% but only contained four studies outside of North America (22).

Aim and objectives

The aim of our systematic review is to provide an up-to-date appraisal of the validity of stroke, ACS and HF diagnoses in adults focused on European EHRs used in primary and secondary care. Our objectives are to:

1. Summarise and pool estimates of the sensitivity, specificity, PPV and negative predictive value (NPV) of stroke, ACS and HF diagnoses compared to a suitable reference standard.
2. Determine whether estimates differ by study population, validation method, data source, diagnosis, and time period.

METHODS

This protocol has been prepared using the Preferred Reporting Items for Systematic Review and Meta-Analysis Protocols guidelines (23).

Eligibility criteria

We used the PICOS (Population, Intervention, Comparator, Outcomes and Study design) framework to formulate the research question and eligibility criteria for our review, but adapted this to replace "Intervention" with "Index test", the modification recommended for systematic reviews of diagnostic test accuracy (24). This modification was chosen as it is the closest resemblance to the validation of EHRs.

Population

Eligible studies will include records of adults aged 16 years or older from any European primary or secondary care national or regional EHR database. We will exclude studies that validate insurance claims or billing databases, disease registries or vital registration systems, as well as studies that validate locally held databases.

Index test

We are interested in records with clinical codes, for example International Classification of Primary Care (ICPC) or International Classifications of Disease (ICD), which identify a diagnosis of stroke, ACS (including MI and other ACS) or HF in primary or secondary care EHRs.

Comparator

To be included, studies must have validated against an internal or external reference standard. Eligible external reference standards include manual review of medical records, patient or clinical questionnaire, or comparison with an independent second database. Internal within database comparison includes validation against a diagnosis algorithm or comparison of clinical codes with anonymized free text.

For peer review only - http://bmjopen.bmj.com/site/about/guidelines.xhtml

Outcome

Studies must either report 1) at least one of the following validation estimates; sensitivity, specificity, PPV and NPV, or 2) data which allows at least validation estimates to be calculated.

Study design

We will include any type of study from any time period published in any language that includes the validation of the recording of stroke, ACS or HF diagnoses in an EHR database, regardless of if this was the main objective of the study.

Information sources

To review published and in-process citations the following databases will be searched from inception to April 2019; MEDLINE, EMBASE, SCOPUS, Web of Science and Cochrane library. Using OpenGrey, and EThOS we will search for relevant grey literature. Bibliographies of national EHR databases used for research will also be searched.

Search strategy

The search strategy will include subject heading terms and free text (title and abstract) for the concept of acute CV events using synonyms of stroke, ACS and HF as well as the concepts of EHRs and validation. We will limit our search to studies conducted using European EHRs. Provisional search terms have been developed for MEDLINE (Appendix 1), and once finalized will be transcribed into corresponding searches for the other aforementioned information sources. We will also review the reference list of other relevant systematic reviews identified during the screening process as well as of articles included in our review to identify further potentially relevant studies.

Study records

Data management

Citations from the searched databases will be exported into Endnote X9. Electronic deduplication of records will be conducted followed by manual deduplication where necessary.

Selection process

For the initial screening stage two authors (JD and RM) will independently review all titles and abstracts to assess whether they fulfil the eligibility criteria for inclusion. To reduce the risk of missing potentially relevant studies, reviewers will adopt a lenient approach for this first level of screening including any study that validate stroke, ACS or HF diagnoses in EHRs. Full-text articles for studies that meet the review criteria will be obtained. The reasons for rejection of articles during the full-text screening process will be noted according to a hierarchical list; 1) could not obtain full text, 2) did not conduct validation, 3) duplicate study, 4) wrong outcome, 5) wrong index (i.e. not a primary or secondary care EHR in Europe), 6) not a suitable comparator, or 7) no validation estimate or insufficient data to calculate. Any discrepancies at either the initial screening or full-text screening will be discussed by the two reviewers, with a third author (CWG) consulted when necessary.

Data collection process

To extract information for each study selected for final inclusion, data extraction tables will be piloted by two authors (JD and RM) for three studies with changes made if required. Data will then be extracted using the finalized template by both authors for five studies. Any discrepancies in the data extracted by

For peer review only - http://bmjopen.bmj.com/site/about/guidelines.xhtml

the two authors will be discussed, and consultation with a third author (CWG) carried out if necessary. Data for each of the remaining studies will then by extracted by a single author (JD).

Data items

Similar to our search strategy, we will use the PICOS framework to systematise the extraction of data from each study. We will use a standardised template containing information on each of the following five domains:

1. Population: participants, age and sex, inclusion and exclusion criteria.
2. Index test: EHR country, EHR name, EHR setting (primary or secondary care), EHR coding system (IPCP, ICD etc.), EHR coverage (regional or national), diagnoses validated including whether incident or prevalent, specific diagnoses codes validated.
3. Comparator: method of validation, description of method.
4. Outcome: number of participant diagnoses planned, number of diagnoses conducted, measures of validity, and raw data to calculate measures of validity.
5. Study characteristics: authors, publication year, language, study design, study period, main aim of the study (validation or not validation).

Outcomes and prioritisation

The outcome is any validation estimate of stroke, ACS or HF. The study has no secondary outcomes.

Risk of bias in individual studies

Two authors (JD and RM) will independently evaluate the risk of bias in five studies, any discrepancies will be discussed and a third author (CWG) consulted when necessary. A single author (JD) will then conduct the risk of bias assessment for each remaining study. To assess bias we will use a tailored version of the Quality Assessment of Diagnostic Accuracy Studies 2 (QUADAS-2) tool, which is used for assessing diagnostic accuracy studies (25), based on previous modifications made for assessing the validity of diagnostic coding in EHRs (19,20). We will consider bias in each of the domains included in QUADAS-2; patient selection, index test, reference standard, and flow and timing. In the context of our review, index test translates to the clinical codes validated. We will produce a summary risk of bias figure, as well as an additional table explaining each judgement.

Data synthesis and meta-bias(es)

We will use a narrative synthesis and forest plots to summarise the validity of each of stroke, ACS and HF diagnoses in European primary and secondary care EHRs. We will consider conducting a meta-analysis if there are studies that are sufficiently homogeneous in terms of population, validation method, data source and outcome. We will use the I^2 statistic to guide judgements about the level of statistical heterogeneity between studies. If there is a sufficient number of studies selected we will explore the reasons for heterogeneity with subgroup analyses by; 1) study populations i.e. specific demographic or clinical groups, 2) validation method, 3) data source i.e. primary care and secondary care EHRs, 4) specific diagnosis i.e. incident or prevalent, and 5) variation in validity estimates over time.

Confidence in cumulative evidence

We will use the Grading of Recommendations, Assessment, Development and Evaluation (GRADE) tool (26) to judge the certainty of cross-study evidence for the validity of diagnoses in EHRs and their use in research. We will examine stroke, MI and HF diagnoses in EHRs for; overall risks of bias, inconsistency, indirectness, imprecision and publication bias with the production of funnel plots. The strength of

For peer review only - http://bmjopen.bmj.com/site/about/guidelines.xhtml

evidence will be categorised as; high, moderate, low and very low. Our judgements will be presented in a summary of findings table.

Patient and public involvement

Patients and or public were not involved in this systematic review.

DISCUSSION

This systematic review will provide an up-to-date assessment of the validity of primary and secondary care EHRs used for stroke, ACS and HF research. This will be beneficial to researchers when considering and deciding whether to use EHRs in their research as well as which diagnostic codes to use. Additionally, the findings will be of benefit in the interpretation and synthesis of results from such studies.

ETHICS AND DISSEMINATION

Ethical review is not required as this study is a systematic review. Important protocol amendments will be documented and a justification for deviating from the original protocol provided in a protocol addendum. The findings of this review will be submitted to a peer-reviewed journal.

Author Contributions CW-G conceived the study idea. JAD lead the design of the study with contributions from AB, LS and CW-G. JAD drafted the methods and analysis and revised the protocol following author comments from CW-G, AB, RM and LS. All authors approved the final version of the protocol.

Funding JAD is supported by a British Heart Foundation PhD Studentship (FS/18/71/33938). The funders have no input on the protocol development and will not have influence on the conduct, analysis, interpretation or publication of the study results.

Conflicts of interest None declared.

REFERENCES

1. World Health Organization. The top 10 causes of death [Internet]. [cited 2019 Feb 11]. Available from: https://www.who.int/en/news-room/fact-sheets/detail/the-top-10-causes-of-death

2. GBD 2016 Stroke Collaborators CO, Nguyen M, Roth GA, Nichols E, Alam T, Abate D, et al. Global, regional, and national burden of stroke, 1990-2016: a systematic analysis for the Global Burden of Disease Study 2016. Lancet Neurol [Internet]. 2019 Mar 11 [cited 2019 Mar 20];0(0). Available from: http://www.ncbi.nlm.nih.gov/pubmed/30871944

3. Evers SMAA, Struijs JN, Ament AJHA, van Genugten MLL, Jager J (Hans) C, van den Bos GAM. International Comparison of Stroke Cost Studies. Stroke [Internet]. 2004 May 1 [cited 2019 Mar 20];35(5):1209–15. Available from: https://www.ahajournals.org/doi/10.1161/01.STR.0000125860.48180.48

4. Saka O, McGuire A, Wolfe C. Cost of stroke in the United Kingdom. Age Ageing [Internet]. 2008 Nov 13 [cited 2019 Mar 20];38(1):27–32. Available from:

For peer review only - http://bmjopen.bmj.com/site/about/guidelines.xhtml

https://academic.oup.com/ageing/article-lookup/doi/10.1093/ageing/afn281

5. Chevreul K, Durand-Zaleski I, Gouépo A, Fery-Lemonnier E, Hommel M, Woimant F. Cost of stroke in France. Eur J Neurol [Internet]. 2013 Jul 1 [cited 2019 Mar 20];20(7):1094–100. Available from: http://doi.wiley.com/10.1111/ene.12143

6. Savarese G, Lund LH. Global Public Health Burden of Heart Failure. Card Fail Rev [Internet]. 2017 Apr [cited 2019 Mar 11];3(1):7–11. Available from: http://www.ncbi.nlm.nih.gov/pubmed/28785469

7. Ambrosy AP, Fonarow GC, Butler J, Chioncel O, Greene SJ, Vaduganathan M, et al. The Global Health and Economic Burden of Hospitalizations for Heart Failure: Lessons Learned From Hospitalized Heart Failure Registries. J Am Coll Cardiol [Internet]. 2014 Apr 1 [cited 2019 Mar 20];63(12):1123–33. Available from: https://www.sciencedirect.com/science/article/pii/S0735109714002915?via%3Dihub

8. Thygesen K, Alpert JS, Jaffe AS, Chaitman BR, Bax JJ, Morrow DA, et al. Fourth universal definition of myocardial infarction (2018). Eur Heart J [Internet]. 2019 Jan 14 [cited 2019 Feb 21];40(3):237–69. Available from: https://academic.oup.com/eurheartj/article/40/3/237/5079081

9. Izzo C, Carrizzo A, Alfano A, Virtuoso N, Capunzo M, Calabrese M, et al. The Impact of Aging on Cardio and Cerebrovascular Diseases. Int J Mol Sci [Internet]. 2018 Feb 6 [cited 2019 Mar 7];19(2). Available from: http://www.ncbi.nlm.nih.gov/pubmed/29415476

10. World Health Organization. Cardiovascular diseases [Internet]. WHO. World Health Organization; 2018 [cited 2019 Mar 7]. Available from: https://www.who.int/cardiovascular_diseases/en/

11. Strike PC, Steptoe A. Behavioral and Emotional Triggers of Acute Coronary Syndromes: A Systematic Review and Critique. Psychosom Med [Internet]. 2005 Mar [cited 2019 Mar 20];67(2):179–86. Available from: http://www.ncbi.nlm.nih.gov/pubmed/15784781

12. Mustafić H, Jabre P, Caussin C, Murad MH, Escolano S, Tafflet M, et al. Main Air Pollutants and Myocardial Infarction. JAMA [Internet]. 2012 Feb 15 [cited 2019 Mar 20];307(7):713. Available from: http://www.ncbi.nlm.nih.gov/pubmed/22337682

13. Estabragh ZR, Mamas MA. The cardiovascular manifestations of influenza: a systematic review. Int J Cardiol [Internet]. 2013 Sep 10 [cited 2019 Feb 11];167(6). Available from: https://linkinghub.elsevier.com/retrieve/pii/S0167527313003537

14. Casey JA, Schwartz BS, Stewart WF, Adler NE. Using Electronic Health Records for Population Health Research: A Review of Methods and Applications. Annu Rev Public Health [Internet]. 2016 Mar 18 [cited 2019 Mar 7];37(1):61–81. Available from: http://www.annualreviews.org/doi/10.1146/annurev-publhealth-032315-021353

15. Doran T, Kontopantelis E, Valderas JM, Campbell S, Roland M, Salisbury C, et al. Effect of financial incentives on incentivised and non-incentivised clinical activities: longitudinal analysis of data from the UK Quality and Outcomes Framework. BMJ [Internet]. 2011 Jun 28 [cited 2019 Mar 20];342:d3590. Available from: http://www.ncbi.nlm.nih.gov/pubmed/21712336

16. Herrett E, Thomas SL, Schoonen WM, Smeeth L, Hall AJ. Validation and validity of diagnoses in the General Practice Research Database: a systematic review. Br J Clin Pharmacol [Internet]. 2010 Jan [cited 2019 Mar 7];69(1):4–14. Available from: http://www.ncbi.nlm.nih.gov/pubmed/20078607

17. Sund R. Quality of the Finnish Hospital Discharge Register: A systematic review. Scand J Public

For peer review only - http://bmjopen.bmj.com/site/about/guidelines.xhtml

Health [Internet]. 2012 Aug 16 [cited 2019 Feb 28];40(6):505–15. Available from: http://journals.sagepub.com/doi/10.1177/1403494812456637

18. Rubbo B, Fitzpatrick NK, Denaxas S, Daskalopoulou M, Yu N, Patel RS, et al. Use of electronic health records to ascertain, validate and phenotype acute myocardial infarction: A systematic review and recommendations. Int J Cardiol [Internet]. 2015 May [cited 2019 Feb 1];187:705–11. Available from: http://www.ncbi.nlm.nih.gov/pubmed/25966015

19. Woodfield R, Grant I, Sudlow CLM, UK Biobank Follow-Up and Outcomes Working Group, Sudlow CLM. Accuracy of Electronic Health Record Data for Identifying Stroke Cases in Large-Scale Epidemiological Studies: A Systematic Review from the UK Biobank Stroke Outcomes Group. Quinn TJ, editor. PLoS One [Internet]. 2015 Oct 23 [cited 2019 Feb 1];10(10):e0140533. Available from: http://www.ncbi.nlm.nih.gov/pubmed/26496350

20. McCormick N, Lacaille D, Bhole V, Avina-Zubieta JA. Validity of Myocardial Infarction Diagnoses in Administrative Databases: A Systematic Review. Guo Y, editor. PLoS One [Internet]. 2014 Mar 28 [cited 2019 Feb 20];9(3):e92286. Available from: https://dx.plos.org/10.1371/journal.pone.0092286

21. McCormick N, Bhole V, Lacaille D, Avina-Zubieta JA. Validity of Diagnostic Codes for Acute Stroke in Administrative Databases: A Systematic Review. Quinn TJ, editor. PLoS One [Internet]. 2015 Aug 20 [cited 2019 Feb 21];10(8):e0135834. Available from: https://dx.plos.org/10.1371/journal.pone.0135834

22. McCormick N, Lacaille D, Bhole V, Avina-Zubieta JA. Validity of Heart Failure Diagnoses in Administrative Databases: A Systematic Review and Meta-Analysis. Guo Y, editor. PLoS One [Internet]. 2014 Aug 15 [cited 2019 Feb 21];9(8):e104519. Available from: http://dx.plos.org/10.1371/journal.pone.0104519

23. Moher D, Shamseer L, Clarke M, Ghersi D, Liberati A, Petticrew M, et al. Preferred reporting items for systematic review and meta-analysis protocols (PRISMA-P) 2015 statement. Syst Rev [Internet]. 2015 Dec 1 [cited 2019 Apr 8];4(1):1. Available from: https://systematicreviewsjournal.biomedcentral.com/articles/10.1186/2046-4053-4-1

24. Centre for Reviews and Dissemination University of York. Systematic Reviews: CRD's guidance for undertaking reviews in health care [Internet]. 2009 [cited 2019 Apr 8]. Available from: https://www.york.ac.uk/media/crd/Systematic_Reviews.pdf

25. Whiting PF, Rutjes AWS, Westwood ME, Mallett S, Deeks JJ, Reitsma JB, et al. QUADAS-2: A Revised Tool for the Quality Assessment of Diagnostic Accuracy Studies. Ann Intern Med [Internet]. 2011 Oct 18 [cited 2019 Mar 12];155(8):529. Available from: http://www.ncbi.nlm.nih.gov/pubmed/22007046

26. Atkins D, Best D, Briss PA, Eccles M, Falck-Ytter Y, Flottorp S, et al. Grading quality of evidence and strength of recommendations. BMJ [Internet]. 2004 Jun 19 [cited 2019 Mar 12];328(7454):1490. Available from: http://www.ncbi.nlm.nih.gov/pubmed/15205295

For peer review only

For peer review only - http://bmjopen.bmj.com/site/about/guidelines.xhtml

Appendix 1. Provisional MEDLINE search terms

1 (Europe or Albania or Andorra or Armenia or Austria or Azerbaijan or Balkan* or Belgium or Belarus or Byelarus or Belorussia or Bosnia or Herzegovia or Bulgaria or Croatia or Cyprus or Czechoslovakia or Czech Republic or Denmark or Faeroe Islands or Estonia or Finland or France or Germany or Great Britain or GBR or United Kingdom or UK or (Wales not New South Wales) or (England not New England) or Northern Ireland or Scotland or Channel Islands or Isle of Man or Greece or Gibraltar or Hungary or Iceland or Ireland or Eire or Italy or Latvia or Liechtenstein or Lithuania or Luxembourg or Kosovo or Macedonia or Malta or Mediterranean or Moldova or Monaco or Montenegro or Netherlands or Holland or Norway or Poland or Portugal or Romania or Russia or Russian Federation or USSR or "Union of Soviet Socialist Republics" or Soviet Union or San Marino or Scandinavia or Serbia or Slovakia or Slovak Republic or Slovenia or Spain or Balearic Islands or Canary Islands or Sweden or Switzerland or Ukraine).ti,ab.
2 turkey.ti,ab. not animal/
3 exp Europe/
4 or/1-3
5 exp Stroke/ or exp Brain Infarction/ or exp Cerebral Hemorrhage/ or exp Subarachnoid Hemorrhage/
6 stroke.ti,ab.
7 cerebrovascular accident.ti,ab.
8 ((brain* or cerebr* or intracerebral or intracran* or subarachnoid) adj (infarct* or thrombo* or emboli* or h?emorrhage or h?ematoma or bleed*)).ti,ab.
9 or/5-8
10 exp Myocardial Infarction/ or Acute Coronary Syndrome/
11 (myocardial infarct* or MI or AMI or acute coronary syndrome or ACS).ti,ab.
12 ((cardiac or heart) adj (infarct* or attack* or arrest* or event*)).ti,ab.
13 (stemi or st-segment or st segment or st-elevat* or st elevat*).ti,ab.
14 (nstemi or non-st-segment or non-st segment or non st segment or non-st-elevat* or non-st elevat* or non st elevat*).ti,ab.
15 or/10-14
16 exp Heart Failure/
17 (heart failure or cardiac failure or CCF or left ventricular failure).ti,ab.
18 (left ventricular adj (systolic or diastolic) adj (dysfunction or impairment)).ti,ab.
19 or/16-18
20 9 or 15 or 19
21 Hospital Records/ or exp Medical Records/
22 ((electronic* or digital* or computer* or longitudinal) adj (health* or medical or clinical or patient) adj (record* or data* or regist*)).ti,ab.
23 (EHR or EPR or EMR or EPD).ti,ab.
24 (routine* collected adj2 data).ti,ab.
25 ((primary care or general practice or secondary care or hospital* or health* or administrative or automated) adj2 (record* or data* or regist*)).ti,ab.
26 exp Clinical Coding/ or exp International Classification of Diseases/
27 ((clinical or medical or read or OXMIS) adj cod*).ti,ab.
28 SNOMED.ti,ab.
29 (International Classification of Diseases or ICD10* or ICD-10* or ICD9* or ICD-9* or International Classification of Primary Care or ICPC).ti,ab.
30 (Clinical Practice Research Datalink or CPRD or General Practice Research Database or GPRD or

For peer review only - http://bmjopen.bmj.com/site/about/guidelines.xhtml

Value Added Medical Products or VAMP or Hospital Episode Statistics or HES or The Health Improvement Network or QResearch or ResearchOne or Danish National Patient Registry or DNPR or Intego or National Patient Register or NPR or patientregistret or National Inpatient Register or IPR or slutenvardsregistret or Finnish Hospital Discharge Register or FHDR or BIFAP or DIRAYA or Dutch National Basic Registration of Hospital Care or Landelijke Basisregistratie Ziekenhuiszorg or LBZ or Dutch Hospital Discharge Register or Landelijke Medische registratie or LMR or Mondriaan or Netherlands Primary Care Research Database or NPCRD or Integrated Primary Care Information or IPCI or Information Network of General Practice or LINH or French Hospital Discharge Database or FHDDB or Health Longitudinal Patient Database or HSD).ti,ab.
31 or/21-30
32 Validation Studies/ or exp Reproducibility of Results/ or exp "sensitivity and specificity"/
33 (sensitivity or specificity or positive predictive value or PPV or negative predictive value or NPV).ti,ab.
34 (accura* or consisten* or reliab* or reproduc* or complet* or precis* or concordance or variation or variab* or replicat* or verif* or valid* or predict*).ti,ab.
35 (ROC or receiver operating characteristic or kappa).ti,ab.
36 or/32-35
37 4 and 20 and 31 and 36

For peer review only

For peer review only - http://bmjopen.bmj.com/site/about/guidelines.xhtml

PRISMA-P (Preferred Reporting Items for Systematic review and Meta-Analysis Protocols) 2015 checklist: recommended items to address in a systematic review protocol*

Section and topic	Item No	Checklist item	Criteria location within manuscript
**ADMINISTRATIVE INFORMATION**			
Title:			
Identification	1a	Identify the report as a protocol of a systematic review	See title & first line of methods
Update	1b	If the protocol is for an update of a previous systematic review, identify as such	Not applicable
Registration	2	If registered, provide the name of the registry (such as PROSPERO) and registration number	See abstract
Authors:			
Contact	3a	Provide name, institutional affiliation, e-mail address of all protocol authors; provide physical mailing address of corresponding author	See title page
Contributions	3b	Describe contributions of protocol authors and identify the guarantor of the review	
Amendments	4	If the protocol represents an amendment of a previously completed or published protocol, identify as such and list changes; otherwise, state plan for documenting important protocol amendments	Not applicable
Support:			
Sources	5a	Indicate sources of financial or other support for the review	See funding section
Sponsor	5b	Provide name for the review funder and/or sponsor	See funding section
Role of sponsor or funder	5c	Describe roles of funder(s), sponsor(s), and/or institution(s), if any, in developing the protocol	See funding section
**INTRODUCTION**			
Rationale	6	Describe the rationale for the review in the context of what is already known	See introduction, paragraphs 1-4
Objectives	7	Provide an explicit statement of the question(s) the review will address with reference to participants, interventions, comparators, and outcomes (PICO)	See introduction, final paragraph
**METHODS**			
Eligibility criteria	8	Specify the study characteristics (such as PICO, study design, setting, time frame) and report characteristics (such as years considered, language, publication status) to be used as criteria for eligibility for the review	See methods section titled “Eligibility criteria”
Information	9	Describe all intended information sources (such as electronic databases, contact with study	See methods section titled “Information sources”

For peer review only

sources		authors, trial registers or other grey literature sources) with planned dates of coverage	
Search strategy	10	Present draft of search strategy to be used for at least one electronic database, including planned limits, such that it could be repeated	See methods section titled "Search strategy"
Study records:			
Data management	11a	Describe the mechanism(s) that will be used to manage records and data throughout the review	See methods section titled "Study records", sub-headed "Data management"
Selection process	11b	State the process that will be used for selecting studies (such as two independent reviewers) through each phase of the review (that is, screening, eligibility and inclusion in meta-analysis)	See methods section titled "Study records", sub-headed "Selection process"
Data collection process	11c	Describe planned method of extracting data from reports (such as piloting forms, done independently, in duplicate), any processes for obtaining and confirming data from investigators	See methods section titled "Study records", sub-headed "Data collection process"
Data items	12	List and define all variables for which data will be sought (such as PICO items, funding sources), any pre-planned data assumptions and simplifications	See methods section titled "Study records", sub-headed "Data items"
Outcomes and prioritization	13	List and define all outcomes for which data will be sought, including prioritization of main and additional outcomes, with rationale	See methods section titled "Outcomes and prioritisation"
Risk of bias in individual studies	14	Describe anticipated methods for assessing risk of bias of individual studies, including whether this will be done at the outcome or study level, or both; state how this information will be used in data synthesis	See methods section titled "Risk of bias in individual studies"
Data synthesis	15a	Describe criteria under which study data will be quantitatively synthesised	See methods section titled "Data synthesis and meta-bias(es)"
	15b	If data are appropriate for quantitative synthesis, describe planned summary measures, methods of handling data and methods of combining data from studies, including any planned exploration of consistency (such as I^2, Kendall's τ)	See methods section titled "Data synthesis and meta-bias(es)"
	15c	Describe any proposed additional analyses (such as sensitivity or subgroup analyses, meta-regression)	See methods section titled "Data synthesis and meta-bias(es)"
	15d	If quantitative synthesis is not appropriate, describe the type of summary planned	Not applicable
Meta-bias(es)	16	Specify any planned assessment of meta-bias(es) (such as publication bias across studies, selective reporting within studies)	See methods section titled "Data synthesis and meta-bias(es)"
Confidence in cumulative evidence	17	Describe how the strength of the body of evidence will be assessed (such as GRADE)	See methods section titled "Confidence in cumulative evidence"

*** It is strongly recommended that this checklist be read in conjunction with the PRISMA-P Explanation and Elaboration (cite when available) for important clarification on the items. Amendments to a review protocol should be tracked and dated. The copyright for PRISMA-P (including checklist) is held by the PRISMA-P Group and is distributed under a Creative Commons Attribution Licence 4.0.**

For peer review only

From: Shamseer L, Moher D, Clarke M, Ghersi D, Liberati A, Petticrew M, Shekelle P, Stewart L, PRISMA-P Group. Preferred reporting items for systematic review and meta-analysis protocols (PRISMA-P) 2015: elaboration and explanation. BMJ. 2015 Jan 2;349(jan02 1):g7647.

For peer review only

BMJ Open

The validity of acute cardiovascular outcome diagnoses in European electronic health records: a systematic review protocol

Journal:	*BMJ Open*
Manuscript ID	bmjopen-2019-031373.R1
Article Type:	Protocol
Date Submitted by the Author:	21-Aug-2019
Complete List of Authors:	Davidson, Jennifer; London School of Hygiene and Tropical Medicine, Faculty of Epidemiology & Population Health<br>Banerjee, Amitava; University College London, Farr Institute of Health Informatics Research<br>Muzambi, Rutendo; London School of Hygiene and Tropical Medicine, Faculty of Epidemiology & Population Health, London<br>Smeeth, Liam; London School of Hygiene and Tropical Medicine, Faculty of Epidemiology & Population Health<br>Warren-Gash, Charlotte; London School of Hygiene and Tropical Medicine, Faculty of Epidemiology & Population Health
**Primary Subject Heading**:	Research methods
Secondary Subject Heading:	Cardiovascular medicine, Epidemiology
Keywords:	electronic health records, validation, systematic review, cardiovascular diseases

SCHOLARONE™
Manuscripts

Title: The validity of acute cardiovascular outcome diagnoses in European electronic health records: a systematic review protocol

Authors: Jennifer A. Davidson[1], Amitava Banerjee[2], Rutendo Muzambi[1], Liam Smeeth[1], Charlotte Warren-Gash[1]

Author affiliations:
1 London School of Hygiene and Tropical Medicine, Faculty of Epidemiology & Population Health, London, UK
2 University College London, Farr Institute of Health Informatics Research, London, UK

Corresponding author details

Name: Jennifer Davidson
Postal address: London School of Hygiene and Tropical Medicine, Room LG21, Keppel Street, London, UK, WC1E 7HT
Email address: Jennifer.Davidson@lshtm.ac.uk
Phone number: +44 (0)20 7636 8636

Key words: electronic health records; cardiovascular diseases; validation; systematic review

Word count: 2,851

For peer review only

Abstract

Introduction Cardiovascular diseases (CVDs) are among the leading causes of death globally. Electronic health records (EHRs) provide a rich data source for research on CVD risk factors, treatments and outcomes. Researchers must be confident in the validity of diagnoses in EHRs, particularly when diagnosis definitions and use of EHRs change over time. Our systematic review provides an up-to-date appraisal of the validity of stroke, acute coronary syndrome (ACS) and heart failure (HF) diagnoses in European primary and secondary care EHRs.

Methods and analysis We will systematically review published and grey literature to identify studies validating diagnoses of stroke, ACS and HF in European EHRs. MEDLINE, EMBASE, SCOPUS, Web of Science, Cochrane library, OpenGrey and EThOS will be searched from dates of inception to April 2019. A prespecified search strategy of subject headings and free text terms in the title and abstract will be used. Two reviewers will independently screen titles and abstracts to identify eligible studies, followed by full-text review. We require studies to compare clinical codes with a suitable reference standard. Additionally, at least one validation measure (sensitivity, specificity, positive predictive value or negative predictive value) or raw data, for the calculation of a validation measure, is necessary. We will then extract data from the eligible studies using standardized tables and assess risk of bias in individual studies using the Quality Assessment of Diagnostic Accuracy Studies 2 (QUADAS-2) tool. Data will be synthesised into a narrative format and heterogeneity assessed. Meta-analysis will be considered when a sufficient number of homogenous studies are available. The overall quality of evidence will be assessed using the Grading of Recommendations, Assessment, Development and Evaluation (GRADE) tool.

Ethics and dissemination This is a systematic review so does not require ethnical approval. Our results will be submitted for peer-review publication.

PROSPERO registration number CRD42019123898

For peer review only - http://bmjopen.bmj.com/site/about/guidelines.xhtml

ARTICLE SUMMARY

Strengths and limitations of this study

- This systematic review will comprehensively evaluate the validity of selected major cardiovascular (CV) diagnoses (stroke, acute coronary syndrome and heart failure) in electronic health record (EHR) databases used in the provision of primary and secondary clinical care in Europe by searching five bibliographic databases and two grey literature sources with no language or date restrictions.
- The Preferred Reporting Items for Systematic Reviews and Meta-Analyses statement will be followed ensuring this systematic review provides high quality scientific results.
- There may be heterogeneity in the results produced by our systematic review due to differences in EHR design and use between countries, in particular the relevance of our findings to countries outside of Europe requires further evaluation.

For peer review only

INTRODUCTION

Rationale

Stroke, acute coronary syndrome (ACS) and heart failure (HF) are three cardiovascular (CV) conditions which substantially contribute to morbidity and mortality. Ischaemic heart disease followed by stroke have been the global leading causes of death for 15 years, and in 2016 accounted for 15.2 million deaths (1). Also in 2016, worldwide more than 13 million people were estimated to have suffered a stroke (2) with healthcare expenditure on stroke estimated to be 3-5% (3–5). An estimated 26 million people are living with HF (6), a chronic condition with acute episodes. HF is estimated to account for 1-2% of healthcare expenditure in Europe and the USA (7). Added to the complication of estimating the burden of CV conditions is changes to definitions; the fourth universal definition of myocardial infarction (MI) was issued in 2018 (8).

Increases in the incidence and prevalence of CV conditions is in part due to an ageing population (9), but also due to modifiable risk factors such as smoking, unhealthful diet and lack of physical exercise, and non-modifiable risk factors such as sex and ethnicity (10). A range of factors such as pollution, infections, emotional stress and physical exertion can also trigger acute CV events particularly in those with pre-existing cardiovascular disease (CVD) (11–13).

Electronic health record (EHR) databases are derived from clinical care records and contain longitudinal patient data on diagnoses, treatment and other clinically relevant variables such as smoking. Administrative databases were developed for financial and management purposes to allocate funding or billing of insurance claims. While both are types of computerized health related data that have been widely used for research, they are quite distinct. In particular, the completeness and accuracy of the morbidity data may differ in the two types of data because of the very different reasons why the data were recorded in the first place. In settings where both clinical and administrative data are available, results from some studies suggest the quality of administrative data is lower (14,15).

High quality EHR based research depends upon correct classification of cases and non-cases. Several systematic reviews have previously appraised the validation of specific European EHRs (16–18) as well as specific conditions recorded within EHRs, including CVD (19–23). The previous systematic reviews on the validity of CVD diagnoses included EHRs, along with administrative databases and vital registration databases. McCormick *et al* reported that the positive predictive value (PPV) of stroke

For peer review only - http://bmjopen.bmj.com/site/about/guidelines.xhtml

diagnosis ranged from 32-98%, with the majority of included studies using administrative data from North America (22), while Woodfield *et al* identified PPVs of >70% for stroke based on results which included a greater proportion of studies from Europe (20), where EHRs are widely used. McCormick *et al*'s review of HF diagnosis validity obtained PPVs ranging from 17-100% but only contained four studies outside of North America (23).

Aim and objectives

The aim of our systematic review is to provide an up-to-date appraisal of the validity of stroke (and its subtypes), ACS (including MI and other ACS) and HF diagnoses in adults focused on European EHRs used in primary and secondary care. Our objectives are to:

1. Summarise and pool estimates of the sensitivity, specificity, PPV and negative predictive value (NPV) of stroke, ACS and HF diagnoses compared to a suitable reference standard.
2. Determine whether estimates differ by study population, validation method, data source, diagnosis, and time period.

METHODS

This protocol has been prepared using the Preferred Reporting Items for Systematic Review and Meta-Analysis Protocols guidelines (24).

Eligibility criteria

We used the PICOS (Population, Intervention, Comparator, Outcomes and Study design) framework to formulate the research question and eligibility criteria for our review, but adapted this to replace "Intervention" with "Index test", the modification recommended for systematic reviews of diagnostic test accuracy (25). This modification was chosen as it is the closest resemblance to the validation of EHRs.

Population

Eligible studies will include records of adults aged 16 years or older from any European primary or secondary care national or regional EHR database. We will exclude studies that validate administrative (insurance claims or billing) databases, disease registries or vital registration systems, as well as studies that validate locally held databases. EHRs and administrative databases collect data for different purposes and may differ in accuracy, we are interested in the validity of EHRs used in clinical settings. The comprehensive data capture methods used to populate disease registries mean these datasets are often used as the gold standard in validation of EHRs so would be unsuitable to include in our validation estimates. Vital registration systems capture CVD deaths, such data can be useful when combined with EHR data but is not by itself of benefit in non-mortality related CVD research. Finally, data from locally held databases are unlikely to be captured in centralised EHRs used in research and therefore validation results are not informative for researchers.

Index test

We are interested in records with clinical codes, for example International Classification of Primary Care (ICPC) or International Classifications of Disease (ICD), which identify a diagnosis of stroke (and its subtypes), ACS (including MI and other ACS) or HF in primary or secondary care EHRs. The ICD-9 and ICD-10 codes we assume studies will include (and which we look to validate) in their stroke, ACS and HF definitions are presented in Table 1.

For peer review only - http://bmjopen.bmj.com/site/about/guidelines.xhtml

For peer review only

Table 1. Provisional list of ICD-9 and ICD-10 codes included in diagnoses of interest

Diagnosis	ICD-9	ICD-10
Stroke	430, 431, 432, 433, 434	I60, I61, I62, I63, I64
Acute coronary syndrome	410, 411	I20.0, I21, I22, I24, I49
Heart failure	428	I11.0, I13.0, I13.2, I50

ICD = International Classifications of Disease

Comparator

To be included, studies must have validated against an internal or external reference standard. Eligible external reference standards include manual review of medical records, patient or clinical questionnaire, or comparison with an independent second database. Internal within database comparison includes validation against a diagnosis algorithm or comparison of clinical codes with anonymized free text.

Outcome

Studies must either report 1) at least one of the following validation estimates; sensitivity, specificity, PPV and NPV, or 2) data which allows at least validation estimates to be calculated.

Study design

We will include any type of study from any time period published in any language that includes the validation of the recording of stroke, ACS or HF diagnoses in an EHR database, regardless of if this was the main objective of the study.

Information sources

To review published and in-process citations the following databases will be searched from inception to April 2019; MEDLINE, EMBASE, SCOPUS, Web of Science and Cochrane library. Using OpenGrey, and EThOS we will search for relevant grey literature. Bibliographies of national EHR databases used for research will also be searched.

Search strategy

The search strategy will include subject heading terms and free text (title and abstract) for the concept of acute CV events using synonyms of stroke, ACS and HF as well as the concepts of EHRs and validation. We will limit our search to studies conducted using European EHRs. Provisional search terms have been developed for MEDLINE (Appendix 1), and once finalized will be transcribed into corresponding searches for the other aforementioned information sources. We will also review the reference list of other relevant systematic reviews identified during the screening process as well as of articles included in our review to identify further potentially relevant studies.

Study records

Data management

Citations from the searched databases will be exported into Endnote X9. Electronic deduplication of records will be conducted followed by manual deduplication where necessary.

Selection process

For the initial screening stage two authors (JD and RM) will independently review all titles and abstracts to assess whether they fulfil the eligibility criteria for inclusion. To reduce the risk of missing potentially

For peer review only - http://bmjopen.bmj.com/site/about/guidelines.xhtml

relevant studies, reviewers will adopt a lenient approach for this first level of screening including any study that validate stroke, ACS or HF diagnoses in EHRs. Full-text articles for studies that meet the review criteria will be obtained and reviewed by the two authors (JD and RM). The reasons for rejection of articles during the full-text screening process will be noted according to a hierarchical list; 1) could not obtain full text, 2) did not conduct validation, 3) duplicate study, 4) wrong outcome, 5) wrong index (i.e. not a primary or secondary care EHR in Europe), 6) not a suitable comparator, or 7) no validation estimate or insufficient data to calculate. Any discrepancies at either the initial screening or full-text screening will be discussed by the two reviewers, with a third author (CWG) consulted when necessary.

Data collection process

To extract information for each study selected for final inclusion, data extraction tables will be piloted by two authors (JD and RM) for three studies with changes made if required. We will then dual extract data from a further 10% of studies using the finalized template. If there are any significant discrepancies between the two reviewers, then we will conduct parallel data extraction for a further 10% of studies, again checking for discrepancies. This process will be repeated until no further discrepancies occur, at which stage the remaining data extraction will be completed by the single reviewer (JD). At each stage a third author (CWG) will be consulted when the two reviewers cannot resolve discrepancies.

Data items

Similar to our search strategy, we will use the PICOS framework to systematise the extraction of data from each study. We will use a standardised template containing information on each of the following five domains:

1. Population: participants, age and sex, inclusion and exclusion criteria.
2. Index test: EHR country, EHR name, EHR setting (primary or secondary care), EHR coding system (IPCP, ICD etc.), EHR coverage (regional or national), diagnoses validated including whether incident or prevalent, specific diagnoses codes validated.
3. Comparator: method of validation, description of method.
4. Outcome: number of participant diagnoses planned, number of diagnoses conducted, measures of validity, and raw data to calculate measures of validity.
5. Study characteristics: authors, publication year, language, study design, study period, main aim of the study (validation or not validation).

Outcomes and prioritisation

The outcome is any validation estimate of stroke (including all subtypes), ACS (MI or other ACS) or HF. The study has no secondary outcomes.

Risk of bias in individual studies

To assess bias we will use a tailored version of the Quality Assessment of Diagnostic Accuracy Studies 2 (QUADAS-2) tool, which is used for assessing diagnostic accuracy studies (26), based on previous modifications made for assessing the validity of diagnostic coding in EHRs (20,21). We will consider bias in each of the domains included in QUADAS-2; patient selection, index test, reference standard, and flow and timing. In the context of our review, index test translates to the clinical codes validated. We will produce a summary risk of bias figure, as well as an additional table explaining each judgement.

Two authors (JD and RM) will independently pilot the tailored QUADAS-2 tool, assessing bias in three of the included studies. Any necessary changes will be made to the tool and dual assessment by the two

For peer review only - http://bmjopen.bmj.com/site/about/guidelines.xhtml

For peer review only

reviewers will be done with the finalized tool for a further 10% of studies. If there are significant discrepancies we will continue parallel risk of bias assessment for another 10% of studies, repeating the process until no further discrepancies occur. Assessment of the remaining studies will be completed by a single reviewer (JD). At each stage a third author (CWG) will be consulted when the two reviewers cannot resolve discrepancies

Data synthesis and meta-bias(es)

We will describe key study characteristics and use a narrative synthesis and forest plots to summarise the validity of each of stroke, ACS and HF diagnoses in European primary and secondary care EHRs. The I^2 statistic will our guide judgements about the level of statistical heterogeneity between studies. We will use the Cochrane suggested guide to grade the heterogeneity as low (0-40%), moderate (30-60%), substantial (50-90%) or considerable (75-100%) obtained from the I^2 statistic (27). If there is a sufficient number of studies selected we will explore the reasons for heterogeneity. We will compare heterogeneity before and after removing studies deemed to be at a high risk of bias overall and by subgroups of; 1) study populations i.e. specific demographic or clinical groups, 2) validation method, 3) data source i.e. primary care and secondary care EHRs, 4) specific diagnosis i.e. incident or prevalent and stroke or ACS subtype, and 5) variation in validity estimates over time.

We will consider conducting meta-analyses for each CV condition to calculate pooled effect estimates for sensitivity, specificity, PPV and NPV if studies are sufficiently homogeneous. Meta-analyses would be conducted by the aforementioned subgroups. Our choice of a fixed or random effects model would also be guided by the level of heterogeneity, with random effects meta-analysis methods followed if there is substantial heterogeneity.

Confidence in cumulative evidence

Two reviewers (JD and RM) will independently use the Grading of Recommendations, Assessment, Development and Evaluation (GRADE) tool (28) to judge the certainty of cross-study evidence for the validity of diagnoses in EHRs and their use in research. Any discrepancies between the two reviewers' judgements will be discussed and resolved, if necessary consulting a third author (CWG). We will examine stroke, MI and HF diagnoses in EHRs for; overall risks of bias, inconsistency, indirectness, imprecision and publication bias with the production of funnel plots. The strength of evidence will be categorised as; high, moderate, low and very low. Our judgements will be presented in a summary of findings table.

Patient and public involvement

Patients and or public were not involved in this systematic review.

DISCUSSION

This systematic review will provide an up-to-date assessment of the validity of primary and secondary care EHRs used for stroke, ACS and HF research. To our knowledge this will be the first systematic review to focus solely on the validity of CVD diagnoses in EHRs. Previous systematic reviews have included EHRs along with administrative databases and vital registration databases. Each of these data sources have a different primary purpose, which in turn will impact the validity of the systems. One previous systematic review of MI diagnoses identified the accuracy for vital registration databases was lower (all PPVs ≤59%) than hospitalization data (three-quarters of studies PPV >59%) (21). However,

For peer review only - http://bmjopen.bmj.com/site/about/guidelines.xhtml
For peer review only

hospitalization grouped EHRs and administrative databases together, so it is unclear if the one-quarter of studies with a PPV ≤59% differed by data source to those with higher PPVs.

Our systematic review will also serve to update several aspects covered by the previous systematic reviews validating CVD diagnoses. McCormick *et al's* 2010 review of MI diagnoses only identified three studies which validated ICD-10 coding (21) while Rubbo *et al's* 2014 review identified eight studies (19). The majority of European countries implemented ICD-10 in the late 1990s. Our search run in April 2019 aims to identify more recent studies validating ICD-10 CVD diagnoses, the results of which are most relevant to today's research. In the majority of studies included in the previous systematic review of HF diagnoses, conducted in 2010, sensitivity was <69% (23). Only one of the three included European studies reported sensitivity, this was 43% (29). With an increase in the prevalence of HF (6), and therefore accompanying public health research, we hypothesise that more studies validating HF diagnoses in EHRs will have been published between 2010 and 2019, the results of which will inform current HF research. Similarly, previous systematic reviews validating stroke diagnoses identified variation in accuracy by stroke subtype (20,22), with the inclusion of up-to-date studies we aim to analyse temporal changes in validity estimates with the assumption that more recent studies should have higher and more consistent estimates across stroke subtype. We also aim to present results for ACS other than MI, such as unstable angina which have not been included in any previous systematic review.

Our systematic review benefits from searching multiple databases with no language barriers, compared to the previous systematic reviews of CVD diagnoses which either only searched one to two databases or only included English language studies. There are some limitations to our systematic review. Firstly, by aiming to validate EHRs, rather than broader computerized health related datasets, we have limited our review to Europe where EHRs operate nationally or covering nationally representative populations with widespread use in research. Consequently, our findings will not be applicable to administrative databases, also commonly used in research. Secondly, our results will not necessarily be applicable to countries outside of Europe using EHRs, if the design and utility of the EHRs differs. Lastly, previous systematic reviews on CVD diagnoses have been unable to conduct meta-analyses due to the level of heterogeneity identified in their results. We hope that by limiting our systematic review to EHRs in Europe, many of which are set up and operated in similar ways, the level of heterogeneity between studies will be reduced. However, we will still be limited by variation in the reference standard used and differences in the codes included in validation. Therefore, it may not be possible to conduct any meta-analysis.

Overall, our systematic review should provide useful and up-to-date findings to inform researchers on the validity of using EHRs in their research.

ETHICS AND DISSEMINATION

Ethical review is not required as this study is a systematic review. Important protocol amendments will be documented and a justification for deviating from the original protocol provided in a protocol addendum. The findings of this review will be submitted to a peer-reviewed journal.

Author Contributions CWG conceived the study idea. JAD lead the design of the study with contributions from AB, LS and CWG. JAD drafted the methods and analysis and revised the protocol following author comments from CWG, AB, RM and LS. All authors approved the final version of the protocol.

Funding JAD is supported by a British Heart Foundation PhD Studentship (FS/18/71/33938). The funders

For peer review only - http://bmjopen.bmj.com/site/about/guidelines.xhtml

have no input on the protocol development and will not have influence on the conduct, analysis, interpretation or publication of the study results.

Conflicts of interest None declared.

REFERENCES

1. World Health Organization. The top 10 causes of death [Internet]. Available from: https://www.who.int/en/news-room/fact-sheets/detail/the-top-10-causes-of-death
2. GBD 2016 Stroke Collaborators CO, Nguyen M, Roth GA, Nichols E, Alam T, Abate D, et al. Global, regional, and national burden of stroke, 1990-2016: a systematic analysis for the Global Burden of Disease Study 2016. Lancet Neurol. 2019 Mar 11;0(0).
3. Evers SMAA, Struijs JN, Ament AJHA, van Genugten MLL, Jager J (Hans) C, van den Bos GAM. International Comparison of Stroke Cost Studies. Stroke. 2004 May 1;35(5):1209–15.
4. Saka O, McGuire A, Wolfe C. Cost of stroke in the United Kingdom. Age Ageing. 2008 Nov 13;38(1):27–32.
5. Chevreul K, Durand-Zaleski I, Gouépo A, Fery-Lemonnier E, Hommel M, Woimant F. Cost of stroke in France. Eur J Neurol. 2013 Jul 1;20(7):1094–100.
6. Savarese G, Lund LH. Global Public Health Burden of Heart Failure. Card Fail Rev. 2017 Apr;3(1):7–11.
7. Ambrosy AP, Fonarow GC, Butler J, Chioncel O, Greene SJ, Vaduganathan M, et al. The Global Health and Economic Burden of Hospitalizations for Heart Failure: Lessons Learned From Hospitalized Heart Failure Registries. J Am Coll Cardiol. 2014 Apr 1;63(12):1123–33.
8. Thygesen K, Alpert JS, Jaffe AS, Chaitman BR, Bax JJ, Morrow DA, et al. Fourth universal definition of myocardial infarction (2018). Eur Heart J. 2019 Jan 14;40(3):237–69.
9. Izzo C, Carrizzo A, Alfano A, Virtuoso N, Capunzo M, Calabrese M, et al. The Impact of Aging on Cardio and Cerebrovascular Diseases. Int J Mol Sci. 2018 Feb 6;19(2).
10. World Health Organization. Cardiovascular diseases [Internet]. WHO. World Health Organization; 2018. Available from: https://www.who.int/cardiovascular_diseases/en/
11. Strike PC, Steptoe A. Behavioral and Emotional Triggers of Acute Coronary Syndromes: A Systematic Review and Critique. Psychosom Med. 2005 Mar;67(2):179–86.
12. Mustafić H, Jabre P, Caussin C, Murad MH, Escolano S, Tafflet M, et al. Main Air Pollutants and Myocardial Infarction. JAMA. 2012 Feb 15;307(7):713.
13. Estabragh ZR, Mamas MA. The cardiovascular manifestations of influenza: a systematic review. Int J Cardiol. 2013 Sep 10;167(6).
14. Laws MB, Michaud J, Shield R, McQuade W, Wilson IB. Comparison of Electronic Health Record-Based and Claims-Based Diabetes Care Quality Measures: Causes of Discrepancies. Health Serv Res. 2018 Aug;53:2988–3006.
15. Shahian DM, Silverstein T, Lovett AF, Wolf RE, Normand S-LT. Comparison of Clinical and Administrative Data Sources for Hospital Coronary Artery Bypass Graft Surgery Report Cards. Circulation. 2007 Mar 27;115(12):1518–27.

For peer review only - http://bmjopen.bmj.com/site/about/guidelines.xhtml

16. Herrett E, Thomas SL, Schoonen WM, Smeeth L, Hall AJ. Validation and validity of diagnoses in the General Practice Research Database: a systematic review. Br J Clin Pharmacol. 2010 Jan;69(1):4–14.

17. Sund R. Quality of the Finnish Hospital Discharge Register: A systematic review. Scand J Public Health. 2012 Aug 16;40(6):505–15.

18. Ludvigsson JF, Andersson E, Ekbom A, Feychting M, Kim J-L, Reuterwall C, et al. External review and validation of the Swedish national inpatient register. BMC Public Health. 2011 Jun 9;11:450.

19. Rubbo B, Fitzpatrick NK, Denaxas S, Daskalopoulou M, Yu N, Patel RS, et al. Use of electronic health records to ascertain, validate and phenotype acute myocardial infarction: A systematic review and recommendations. Int J Cardiol. 2015 May;187:705–11.

20. Woodfield R, Grant I, Sudlow CLM, UK Biobank Follow-Up and Outcomes Working Group, Sudlow CLM. Accuracy of Electronic Health Record Data for Identifying Stroke Cases in Large-Scale Epidemiological Studies: A Systematic Review from the UK Biobank Stroke Outcomes Group. Quinn TJ, editor. PLoS One. 2015 Oct 23;10(10):e0140533.

21. McCormick N, Lacaille D, Bhole V, Avina-Zubieta JA. Validity of Myocardial Infarction Diagnoses in Administrative Databases: A Systematic Review. Guo Y, editor. PLoS One. 2014 Mar 28;9(3):e92286.

22. McCormick N, Bhole V, Lacaille D, Avina-Zubieta JA. Validity of Diagnostic Codes for Acute Stroke in Administrative Databases: A Systematic Review. Quinn TJ, editor. PLoS One. 2015 Aug 20;10(8):e0135834.

23. McCormick N, Lacaille D, Bhole V, Avina-Zubieta JA. Validity of Heart Failure Diagnoses in Administrative Databases: A Systematic Review and Meta-Analysis. Guo Y, editor. PLoS One. 2014 Aug 15;9(8):e104519.

24. Moher D, Shamseer L, Clarke M, Ghersi D, Liberati A, Petticrew M, et al. Preferred reporting items for systematic review and meta-analysis protocols (PRISMA-P) 2015 statement. Syst Rev. 2015 Dec 1;4(1):1.

25. Centre for Reviews and Dissemination University of York. Systematic Reviews: CRD's guidance for undertaking reviews in health care [Internet]. 2009.

26. Whiting PF, Rutjes AWS, Westwood ME, Mallett S, Deeks JJ, Reitsma JB, et al. QUADAS-2: A Revised Tool for the Quality Assessment of Diagnostic Accuracy Studies. Ann Intern Med. 2011 Oct 18;155(8):529.

27. The Cochrane Collaboration. Cochrane Handbook for Systematic Reviews of Interventions [Internet]. Version 5. Higgins JP, Green S, editors. 2011.

28. Atkins D, Best D, Briss PA, Eccles M, Falck-Ytter Y, Flottorp S, et al. Grading quality of evidence and strength of recommendations. BMJ. 2004 Jun 19;328(7454):1490.

29. Merry AHH, Boer JMA, Schouten LJ, Feskens EJM, Verschuren WMM, Gorgels APM, et al. Validity of coronary heart diseases and heart failure based on hospital discharge and mortality data in the Netherlands using the cardiovascular registry Maastricht cohort study. Eur J Epidemiol. 2009 May 1;24(5):237–47.

For peer review only

For peer review only - http://bmjopen.bmj.com/site/about/guidelines.xhtml

Appendix 1. Provisional MEDLINE search terms

1 (Europe or Albania or Andorra or Armenia or Austria or Azerbaijan or Balkan* or Belgium or Belarus or Byelarus or Belorussia or Bosnia or Herzegovia or Bulgaria or Croatia or Cyprus or Czechoslovakia or Czech Republic or Denmark or Faeroe Islands or Estonia or Finland or France or Germany or Great Britain or GBR or United Kingdom or UK or (Wales not New South Wales) or (England not New England) or Northern Ireland or Scotland or Channel Islands or Isle of Man or Greece or Gibraltar or Hungary or Iceland or Ireland or Eire or Italy or Latvia or Liechtenstein or Lithuania or Luxembourg or Kosovo or Macedonia or Malta or Mediterranean or Moldova or Monaco or Montenegro or Netherlands or Holland or Norway or Poland or Portugal or Romania or Russia or Russian Federation or USSR or "Union of Soviet Socialist Republics" or Soviet Union or San Marino or Scandinavia or Serbia or Slovakia or Slovak Republic or Slovenia or Spain or Balearic Islands or Canary Islands or Sweden or Switzerland or Ukraine).ti,ab.
2 turkey.ti,ab. not animal/
3 exp Europe/
4 or/1-3
5 exp Stroke/ or exp Brain Infarction/ or exp Cerebral Hemorrhage/ or exp Subarachnoid Hemorrhage/ 6 stroke.ti,ab.
7 cerebrovascular accident.ti,ab.
8 ((brain* or cerebr* or intracerebral or intracran* or subarachnoid) adj (infarct* or thrombo* or emboli* or h?emorrhage or h?ematoma or bleed*)).ti,ab.
9 or/5-8
10 exp Myocardial Infarction/ or Acute Coronary Syndrome/
11 (myocardial infarct* or MI or AMI or acute coronary syndrome or ACS).ti,ab.
12 ((cardiac or heart) adj (infarct* or attack* or arrest* or event*)).ti,ab.
13 (stemi or st-segment or st segment or st-elevat* or st elevat*).ti,ab.
14 (nstemi or non-st-segment or non-st segment or non st segment or non-st-elevat* or non-st elevat* or non st elevat*).ti,ab.
15 or/10-14
16 exp Heart Failure/
17 (heart failure or cardiac failure or CCF or left ventricular failure).ti,ab.
18 (left ventricular adj (systolic or diastolic) adj (dysfunction or impairment)).ti,ab.
19 or/16-18
20 9 or 15 or 19
21 Hospital Records/ or exp Medical Records/
22 ((electronic* or digital* or computer* or longitudinal) adj (health* or medical or clinical or patient) adj (record* or data* or regist*)).ti,ab.
23 (EHR or EPR or EMR or EPD).ti,ab.
24 (routine* collected adj2 data).ti,ab.
25 ((primary care or general practice or secondary care or hospital* or health* or administrative or automated) adj2 (record* or data* or regist*)).ti,ab.
26 exp Clinical Coding/ or exp International Classification of Diseases/
27 ((clinical or medical or read or OXMIS) adj cod*).ti,ab.
28 SNOMED.ti,ab.
29 (International Classification of Diseases or ICD10* or ICD-10* or ICD9* or ICD-9* or International Classification of Primary Care or ICPC).ti,ab.
30 (Clinical Practice Research Datalink or CPRD or General Practice Research Database or GPRD or Value Added Medical Products or VAMP or Hospital Episode Statistics or HES or The Health Improvement Network or QResearch or ResearchOne or Danish National Patient Registry or DNPR or Intego or National Patient Register or NPR or patientregistret or National Inpatient Register or IPR or slutenvardsregistret or Finnish Hospital Discharge Register or FHDR or BIFAP or DIRAYA or

For peer review only

Dutch National Basic Registration of Hospital Care or Landelijke Basisregistratie Ziekenhuiszorg or LBZ or Dutch Hospital Discharge Register or Landelijke Medische registratie or LMR or Mondriaan or Netherlands Primary Care Research Database or NPCRD or Integrated Primary Care Information or IPCI or Information Network of General Practice or LINH or French Hospital Discharge Database or FHDDB or Health Longitudinal Patient Database or HSD).ti,ab.

31 or/21-30

32 Validation Studies/ or exp Reproducibility of Results/ or exp "sensitivity and specificity"/

33 (sensitivity or specificity or positive predictive value or PPV or negative predictive value or NPV).ti,ab.

34 (accura* or consisten* or reliab* or reproduc* or complet* or precis* or concordance or variation or variab* or replicat* or verif* or valid* or predict*).ti,ab.

35 (ROC or receiver operating characteristic or kappa).ti,ab.

36 or/32-35

37 4 and 20 and 31 and 36

For peer review only

PRISMA-P (Preferred Reporting Items for Systematic review and Meta-Analysis Protocols) 2015 checklist: recommended items to address in a systematic review protocol*

Section and topic	Item No	Checklist item	Criteria location within manuscript
**ADMINISTRATIVE INFORMATION**			
Title:			
Identification	1a	Identify the report as a protocol of a systematic review	See title & first line of methods
Update	1b	If the protocol is for an update of a previous systematic review, identify as such	Not applicable
Registration	2	If registered, provide the name of the registry (such as PROSPERO) and registration number	See abstract
Authors:			
Contact	3a	Provide name, institutional affiliation, e-mail address of all protocol authors; provide physical mailing address of corresponding author	See title page
Contributions	3b	Describe contributions of protocol authors and identify the guarantor of the review	
Amendments	4	If the protocol represents an amendment of a previously completed or published protocol, identify as such and list changes; otherwise, state plan for documenting important protocol amendments	Not applicable
Support:			
Sources	5a	Indicate sources of financial or other support for the review	See funding section
Sponsor	5b	Provide name for the review funder and/or sponsor	See funding section
Role of sponsor or funder	5c	Describe roles of funder(s), sponsor(s), and/or institution(s), if any, in developing the protocol	See funding section
**INTRODUCTION**			
Rationale	6	Describe the rationale for the review in the context of what is already known	See introduction, paragraphs 1-4
Objectives	7	Provide an explicit statement of the question(s) the review will address with reference to participants, interventions, comparators, and outcomes (PICO)	See introduction, final paragraph
**METHODS**			
Eligibility criteria	8	Specify the study characteristics (such as PICO, study design, setting, time frame) and report characteristics (such as years considered, language, publication status) to be used as criteria for eligibility for the review	See methods section titled "Eligibility criteria"
Information	9	Describe all intended information sources (such as electronic databases, contact with study	See methods section titled "Information sources"

For peer review only

sources		authors, trial registers or other grey literature sources) with planned dates of coverage	
Search strategy	10	Present draft of search strategy to be used for at least one electronic database, including planned limits, such that it could be repeated	See methods section titled "Search strategy"
Study records:			
Data management	11a	Describe the mechanism(s) that will be used to manage records and data throughout the review	See methods section titled "Study records", sub-headed "Data management"
Selection process	11b	State the process that will be used for selecting studies (such as two independent reviewers) through each phase of the review (that is, screening, eligibility and inclusion in meta-analysis)	See methods section titled "Study records", sub-headed "Selection process"
Data collection process	11c	Describe planned method of extracting data from reports (such as piloting forms, done independently, in duplicate), any processes for obtaining and confirming data from investigators	See methods section titled "Study records", sub-headed "Data collection process"
Data items	12	List and define all variables for which data will be sought (such as PICO items, funding sources), any pre-planned data assumptions and simplifications	See methods section titled "Study records", sub-headed "Data items"
Outcomes and prioritization	13	List and define all outcomes for which data will be sought, including prioritization of main and additional outcomes, with rationale	See methods section titled "Outcomes and prioritisation"
Risk of bias in individual studies	14	Describe anticipated methods for assessing risk of bias of individual studies, including whether this will be done at the outcome or study level, or both; state how this information will be used in data synthesis	See methods section titled "Risk of bias in individual studies"
Data synthesis	15a	Describe criteria under which study data will be quantitatively synthesised	See methods section titled "Data synthesis and meta-bias(es)"
	15b	If data are appropriate for quantitative synthesis, describe planned summary measures, methods of handling data and methods of combining data from studies, including any planned exploration of consistency (such as I^2, Kendall's τ)	See methods section titled "Data synthesis and meta-bias(es)"
	15c	Describe any proposed additional analyses (such as sensitivity or subgroup analyses, meta-regression)	See methods section titled "Data synthesis and meta-bias(es)"
	15d	If quantitative synthesis is not appropriate, describe the type of summary planned	Not applicable
Meta-bias(es)	16	Specify any planned assessment of meta-bias(es) (such as publication bias across studies, selective reporting within studies)	See methods section titled "Data synthesis and meta-bias(es)"
Confidence in cumulative evidence	17	Describe how the strength of the body of evidence will be assessed (such as GRADE)	See methods section titled "Confidence in cumulative evidence"

*** It is strongly recommended that this checklist be read in conjunction with the PRISMA-P Explanation and Elaboration (cite when available) for important clarification on the items. Amendments to a review protocol should be tracked and dated. The copyright for PRISMA-P (including checklist) is held by the PRISMA-P Group and is distributed under a Creative Commons Attribution Licence 4.0.**

For peer review only

From: Shamseer L, Moher D, Clarke M, Ghersi D, Liberati A, Petticrew M, Shekelle P, Stewart L, PRISMA-P Group. Preferred reporting items for systematic review and meta-analysis protocols (PRISMA-P) 2015: elaboration and explanation. BMJ. 2015 Jan 2;349(jan02 1):g7647.

For peer review only
